# The Alignment of Agent-First Preferences with Visual Event Representations: Contrasting German and Arabic

**DOI:** 10.1007/s10936-020-09750-3

**Published:** 2021-03-11

**Authors:** Yulia Esaulova, Sarah Dolscheid, Sabine Reuters, Martina Penke

**Affiliations:** 1 Department of Special Education and Rehabilitation, University of Cologne, Herbert-Lewin-Str. 10, Cologne, 50931 Germany; 2 Department of Psychology, University of Bielefeld, Bielefeld, Germany

**Keywords:** Agent-first preference, Visual event representations, Word order, Argument structure, Writing direction

## Abstract

How does non-linguistic, visual experience affect language production? A series of experiments addressed this question by examining linguistic and visual preferences for agent positions in transitive action scenarios. In Experiment 1, 30 native German speakers described event scenes where agents were positioned either to the right or to the left of patients. Produced utterances had longer speech onset times for scenes with right- rather than left-positioned agents, suggesting that the visual organization of events can affect sentence production. In Experiment 2 another cohort of 36 native German participants indicated their aesthetic preference for left- or right-positioned agents in mirrored scenes and displayed a preference for scenes with left-positioned agents. In Experiment 3, 37 Arabic native participants performed the same non-verbal task showing the reverse preference. Our findings demonstrate that non-linguistic visual preferences seem to affect sentence production, which in turn may rely on the writing system of a specific language.

## Introduction

The intricate relationship between the visual world and its symbolic representation in language provides one of the prime examples for the interaction between linguistic and cognitive systems. In particular, visual properties of a scene (e.g., the salience of an object) can affect the way speakers produce an utterance. For instance, when asked to describe a video clip in which a blue fish ate a red fish, speakers’ utterances were affected by a visual cue, i.e. an arrow pointing to the red fish (Tomlin 1995). Once the red fish was visually highlighted, participants started their utterances by naming the patient first, producing more passive voice sentences (*A red fish is eaten by a blue fish*) than agent-first active voice structures (*A blue fish is eating a red fish*) (Tomlin 1995; also see Tomlin 1997; Myachykov et al. 2007, 2018). These findings suggest that visual salience can affect how speakers formulate a sentence. Converging evidence comes from studies in which the visual salience of an agent was reduced (e.g., Rissman et al. 2018). That is, when the agent of a visual scene was partially occluded and thus perceptually less salient, speakers tended to start their utterances with the patient rather than with the agent, leading to a higher proportion of passive sentences (Rissman et al. 2018). As these examples indicate, visual properties of a scene can affect sentence choice, supporting the notion of close links between vision and linguistic structure.

The present paper seeks to closer examine the relationship between visual and linguistic structure by focusing on the relative positioning of referents in visually depicted transitive events. While a number of studies have established that people have spatial preferences when they listen to transitive event descriptions (e.g., they prefer agents positioned to the left of patients, Chatterjee et al. 1999), here we focus on language production: to what extent does the visuo-spatial position of a referent (i.e., whether a patient of an action is depicted to the right or to the left of the agent) affect how speakers encode a scene and how they produce an utterance? To answer this question, we do not only focus on speakers’ syntactic choices but also measure their speech onset times as a more direct indication of sentence planning. We likewise examine whether potential visual preferences are susceptible to cross-cultural variation by investigating German participants who use a left-to-right writing system as well as Arabic participants who are literate in Modern Standard Arabic (a language with a right-to-left writing system).

In the real world, visual events are structured and perceived along both spatial and temporal axes. The spatial aspect consists in the location of the participants of the action, such as the agent and the patient of an action, relative to one another. The temporal aspect comprises the realization of the action itself starting with the agent’s activity and ending with the patient’s transformation. Word order phenomena represent such an organization of events in language, allowing for both spatial linearity and temporal sequencing on the sentence level. The spatial structuring becomes evident in the written modality of language, where agents are placed in sentence-initial positions and are followed by patients (active sentences in languages with subject-object word order). Depending on the writing system of a language, this spatial structure can be realized in a left-to-right (e.g., German) or right-to-left (e.g., Standard Modern Arabic) manner. The temporal sequencing is reflected in the spoken modality of language as constituents of an utterance are produced one after another as speech unfolds in time. When language users interact with the real world, visual input (e.g., location of actants) and its linguistic realization need to be integrated. To describe a transitive event that unfolds in space and time, one has to bring its elements (e.g., agent, patient and action) into a linear order, making decisions about where to place them in a sentence. These decisions may be influenced by both speakers’ preferences in the perception of visual events and in the linearization of sentence constituents. The extent to which speakers’ preferences in visual and linguistic modalities may interact was one of the central questions of this paper.

### Agent-First Preference

Typological studies have identified a cross-linguistically predominant pattern for the sequential representation of events in languages: subjects/agents precede objects/patients, such as in subject-verb-object and subject-object-verb word orders (e.g., English and German), as well as in verb-subject-object word order (e.g., Arabic) (e.g., Dryer 2013; Kemmerer 2012). The principle of subject salience is often claimed to explain the syntactic organization of these word orders, where a subject occurs before an object in a sentence (e.g., Tomlin 1986; Song 1991). According to this principle, syntactic functions differ in salience with subjects being most salient. While the subject-object hierarchy relates to arguments in a sentence on a syntactic level, it is often mapped onto the hierarchy of thematic roles such as agent and patient, thus establishing a connection between syntax and semantics (Himmelmann and Primus 2015). Psycholinguistic research has repeatedly demonstrated that the linear ordering of syntactic functions (subjects and objects) and thematic roles (agents and patients) is not only reflected in cross-linguistic patterns where subjects/agents precede objects/patients in word order but also has important cognitive implications for language users in that violations of the subject/agent-first preference are associated with more costs and comprehension errors. Ferreira (2003), for instance, reported that participants made more mistakes when they were asked to identify agents and patients in passive sentences where the patient was realized as the subject compared to active sentences where the agent was the subject. Similarly, relative clauses that start with a patient (object-extracted relative clauses, such as *Die Studenten, die die Fahrradfahrerin übersehen hat, sind verletzt* ‘The students, whom the cyclist overlooked, are hurt’) are reported as more difficult for comprehension than those that start with an agent (subject-extracted relative clauses, such as *Die Studenten, die die Fahrradfahrerin übersehen haben, sind verletzt* ‘The students, who overlooked the cyclist, are hurt’) (e.g., Friederici et al. 1998; Gordon et al. 2001; Staub 2010; Esaulova et al. 2017).

### Linguistic Serialization and Visual Event Representations

People not only seem to prefer sequential agent-first structures in language but they also seem to have spatial preferences for agents in visual event scenes. For instance, Chatterjee et al. (1999) observed that agents were located to the left of patients when they asked participants to draw depictions of simple transitive sentences like *The circle hits the square*. In a picture-matching task, participants were faster in matching auditorily presented transitive sentences (e.g., *The circle pushes the square*) to pictures that included a left-positioned agent, suggesting that the linear preference to start a sentence with the agent/subject may find a correspondence in the spatial representation of actants in visual representations where agents are preferred to the left of patients. Converging evidence for this finding comes from a study by Dobel et al. (2007a). In a set of experiments, German-speaking participants were asked to identify the characters depicted in a visual scene that was presented for a very short duration (ranging from 100 to 300 ms). Participants were better at identifying the respective characters when the agent was positioned to the left of the patient rather than the reverse. These findings lend further support to the idea that participants preferably encode transitive events from left to right. While Chatterjee et al. (1999) assume that the left-to-right bias in visual event representations may be due to universal factors like hemispheric asymmetries in brain organization, other findings have challenged this assumption by stressing the impact of cultural factors related to reading and/or writing direction (e.g., Dobel et al. 2007b, 2014; Maass & Russo 2003; Maass et al. 2014; Butler et al. 2014).

### The Influence of Reading/Writing Direction

Studies suggest that visual preferences as observed in the tasks described above relate to the reading/writing direction in language users’ native languages. When testing speakers of Italian and Arabic in similar drawing and picture-matching tasks as Chatterjee et al. (1999), Italian-speakers displayed a left-to-right preference for agent placement, but speakers of Arabic preferred agents on the right of the patient (Maass and Russo 2003, Maass et al. 2014, however see Altmann et al. 2006 who did not observe any differences between English- and Arabic-speaking participants). Dobel et al. (2007b) tested adult and child speakers of German, a language with a left-to-right writing system, and Hebrew, a language with a right-to-left writing system, to find out whether and how literacy has an influence on preferences in the placement of thematic roles. Participants heard sentences containing an agent, an object and a recipient (e.g., *The mother gives the boy a ball*) and had to arrange the depictions of these three noun phrases linearly to match the content of the sentences. As expected, German-speaking adults showed a preference for placing agents on the left side of the pictures of the other two arguments, whereas Hebrew-speaking adults preferably placed the agent to the right of the other arguments. In contrast, preliterate German and Hebrew children displayed no spatial preference, suggesting that the preference with respect to the directionality of a visual event representation is introduced by learning how to read and write. In a similar vein, Dobel et al. (2014) found that illiterate speakers of Yucatec and Spanish revealed no spatial preference when they were asked to draw events, suggesting that literacy can indeed affect the spatial structure of event representation.

**Fig. 1:**
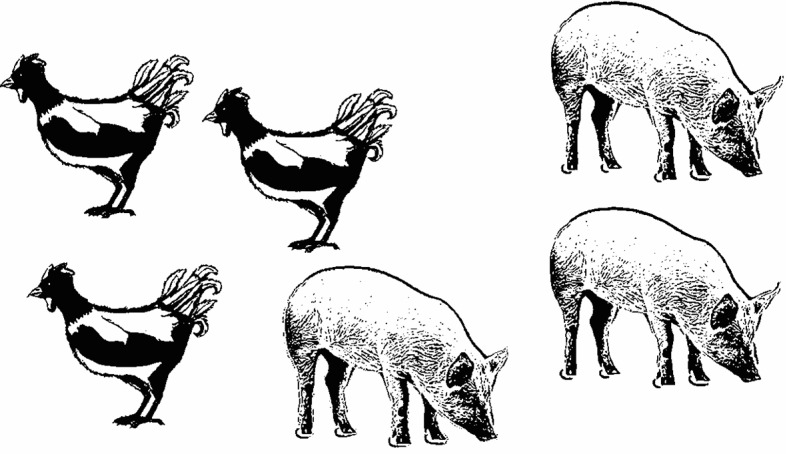
Illustration of the stimuli used by Butler et al. (2014)

The studies mentioned above suggest that visual preferences in depicting an event scene may be associated with habits that are shaped by the characteristics of a writing system. While studies so far have addressed the agent-left preference in visual event representation in tasks involving language comprehension, it remains unclear whether and how these visual preferences will manifest in contexts that involve language production. There is some first evidence provided by Butler et al. (2014) that left-to-right preferences for visual event representations appear to influence utterance structures. In their study, Spanish-speaking and Yucatec-speaking participants who were also fluent in Spanish were asked to describe pictures verbally by replacing a more general term like *the animals* with the particular types they saw on a picture (i.e., the chicken and the pigs, see Fig. 1), thus producing either a sentence like *The chicken and the pigs are eating* (left–right) or *The pigs and the chicken are eating* (right-left).

Monolingual Spanish-speaking participants displayed a left preference, predominantly starting their utterances with the left-most objects, animals, or entities in the picture. Bilingual Yucatec-Spanish speakers showed the same preference in Spanish but no such preference in Yucatec. The authors conclude that speakers of two languages who are literate in one language (i.e., Spanish) but not the other (i.e., Yucatec) show the effect of left-to-right sequencing only in the language they are literate in. The study indicates that not only reading habits in general but the literacy in a particular language affects structural preferences. Importantly, however, while these findings shed some first light on the effect of visual preferences on language production, they do not cover transitive event scenarios that include agent-patient interactions, and thus do not address the issue of how the visual positioning of agent and patient influences sentence production. The present study sought to fill this gap by examining whether the positioning of event characters has an effect on speakers’ event descriptions. Beyond focusing on word order, we also assessed speakers’ speech onset times as a measure tapping into the process of sentence planning.

Even though investigations of language production that did use picture materials with transitive event representations have quite a long tradition in psycholinguistic research, they mainly focused on the effects of structural priming (e.g., Bock et al. 2004). Typically, the employed paradigm has participants listen to an active or a passive sentence before they view and describe a picture. While some of these studies do mention preferences for left-positioned agents on priming effects (Bock 1986; Hartsuiker and Kolk 1998), it remains unclear to which extent spontaneous picture descriptions in relatively natural language settings may be affected by the spatial arrangement of the actants involved in a visually represented event. Our study attempts to shed light on this issue, thus contributing to the research on the interface of event representations and linguistic preferences.

### Overview of Experiments and Hypotheses

The methodological approach of the present study comprises non-verbal and verbal tasks in a series of experiments, which allows assessing both visual preferences for transitive event representations and their potential effects on sentence production.

Experiment 1 was designed to provide information on the issue whether the spatial structure of presented event scenes in a picture-description task affects the choice of syntactic structure and the relative ease or difficulty of sentence production as estimated by speech onset latencies. In German, subject-verb-object word order with a sentence-initial agent is the predominant, canonical word order in main clauses (e.g., *Der Taucher schlägt den Zwerg* ‘The_NOM_ diver_AGENT_ is hitting the_ACC_ dwarf_PATIENT_’). Patients may also appear in sentence-initial positions in German, for instance when a passive sentence (e.g., *Der Zwerg wird vom Taucher geschlagen* ‘The_NOM_ dwarf_PATIENT_ is hit by the_DAT_ diver_AGENT_’) or a sentence with a topicalized patient is constructed (e.g., *Den Zwerg schlägt der Taucher* ‘The_ACC_ dwarf_PATIENT_ hits the_NOM_ diver_AGENT_’). Both sentence types are, however, more marked and less frequent compared to the canonic subject-verb-object order. If the linear organization of visually represented transitive event scenes does affect the syntactic structure of sentences, one should observe differences in language production when participants have to describe scenes depicting agents to the left as opposed to scenes depicting agents to the right of patients. These differences may be expressed in the syntactic choice participants make. Thus we might expect a larger number of non-canonical patient-first sentence constructions, such as passive structures or object topicalizations, to describe scenes with a left-positioned patient. We might also expect an effect in speech onset times, i.e., longer speech onset times when scenes with a left-positioned patient have to be described.

Experiment 2 complemented Experiment 1 in that it examined whether the expected interplay between the visual position of an agent and the agent-first preference in sentence production, targeted in Experiment 1, indeed relates to a non-linguistic factor, i.e. a visual preference for a left-positioned agent in event depiction. To test this hypothesis, stimuli similar to those used in Experiment 1 were used in Experiment 2 but in a non-verbal picture-preference task. In particular, we made use of an aesthetic judgment task where participants simply had to indicate which of two pictures they liked better. While previous work has shown that people display spatial biases in their aesthetic preferences (e.g. regarding a left-to-right arrangement of geometric figures, Christman and Pinger 1997, also see Nachson et al. 1999), non-linguistic aesthetic judgment tasks have not yet been applied to event scenes depicting transitive events. Furthermore, whereas a number of studies have demonstrated that speakers display spatial biases when motor actions are involved (e.g. by drawing event characters or by sorting/arranging images of referents), our goal was to administer a task devoid of a potential confound exerted by such motor actions. Our prediction was as follows: If the agent-first strategy in sentence production relates to a visual preference in event depiction, the preference for a left-situated agent should also surface in our non-verbal aesthetic judgment task.

Experiment 3 follows up on the first two experiments and addressed the question of whether agent-left preferences are subject to cross-cultural variation and driven by the writing system of a given language. If this is the case, we should observe an opposite pattern of preferences (i.e., agent-right) in a language with a right-to-left writing system, such as Modern Standard Arabic, compared to a language with a left-to-right writing system, such as German. Unlike previous studies that encouraged participants to spatialize transitive events (e.g. by means of drawing, Dobel et al. 2014; Maass and Russo 2003), we were interested in whether cross-cultural differences in event representation also surfaced in a non-verbal aesthetic judgment task.

## Experiment 1

Experiment 1 addressed the question of whether sentence production is affected by the positioning of actants in visual depictions of transitive event scenes that are to be described.

### Method

#### Participants

Thirty native speakers of German (25 female, 5 male, mean age 22.6 years, *SD *= 2.3), most of whom were students at the University of Cologne, were paid 8 € to participate in the experiment. None of them reported to be a native speaker of another language in addition to German. All of them had normal or corrected vision and hearing and no health conditions that could affect visual perception or speech production.

### Materials

#### Experimental Stimuli

**Fig. 2:**
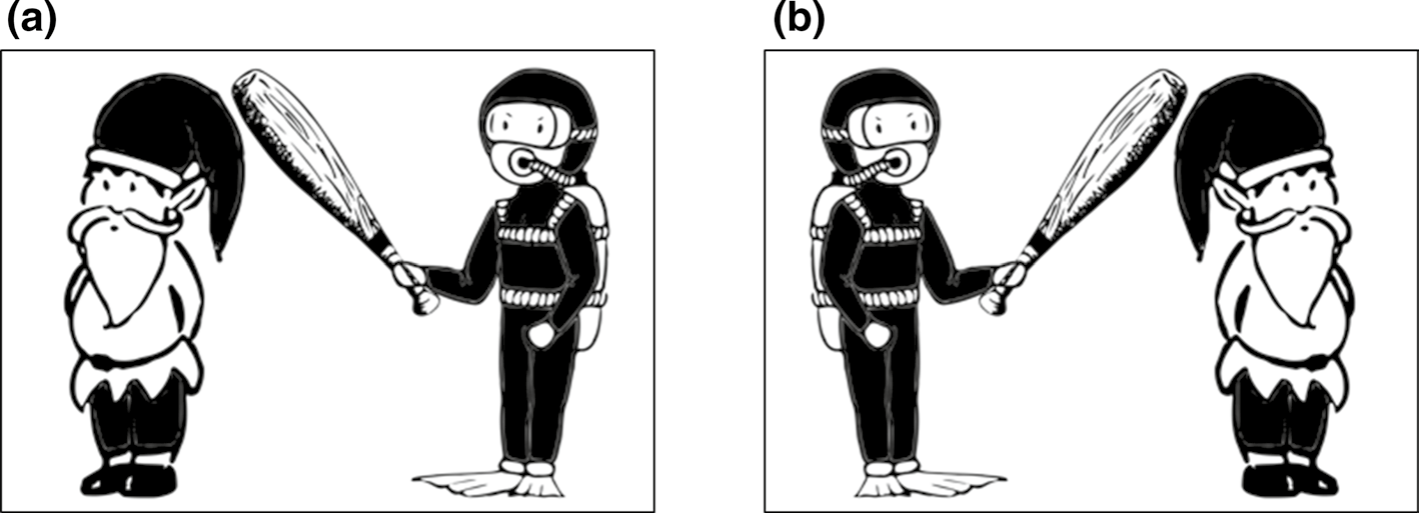
An example of an agent positioned to the right (**a**) and to the left (**b**) of a patient

The experimental stimuli consisted of 32 black-and-white drawings showing simple interactions between an agent and a patient. On 16 of these pictures, the agent was positioned on the right and the patient on the left, whereas on the other half the arrangement of actants was vice versa (see Fig. 2).

Only figures corresponding to grammatically masculine nouns were depicted to control for effects of different morphological case markings in German. Depicted nouns were controlled for syllable length (one to two syllables) and lemma frequency.^1^ The depictions of agent and patient actants did not differ in size and visual complexity across items.

#### Fillers

Thirty-two filler items were constructed to assure that the production of picture descriptions was not influenced by strategies (e.g., the repetition of the same syntactic structure) developed by participants. 16 of these filler stimuli were meant to elicit intransitive sentences, such as *Die Frau weint* ‘The woman is crying’, whereas 16 further pictures showed different arrangements of diverse geometrical shapes that were intended to evoke locative sentences, as in *Das Herz ist neben dem Stern* ‘The heart is next to the star’.

#### Procedure

The experiment was performed using *Presentation*^*®*^ *software* (Presentation 2017), while the produced utterances were recorded. First, a fixation cross appeared for 600 ms in the center of the screen. After this, an event scene was displayed for 7 s, during which participants produced a description of the scene in one sentence. For measuring speech onset times by means of the phonetic analysis tool *Praat* (Boersma and Weenink 2017) and further chronometric analysis, the appearance of each picture on the screen was accompanied by an audio signal.

Participants were asked to describe the presented pictures in a single sentence as quickly as possible. No examples of utterances were provided in the instruction to avoid structural priming of the participants to a particular sentence structure such as active, passive or topicalized sentences. Participants were familiarized with the task by describing 14 pictures similar to those presented later in the experiment in one sentence and received a corrective feedback from the experimenter if an inadequate description of an event scene was provided (e.g., simply naming characters or objects depicted in the picture instead of describing the event in a single sentence (e.g. *ein Teufel* ‘a devil’ instead of *Der Teufel trägt einen Sack* ‘the devil is carrying a bag.’). Each participant received one of the two lists that presented items in a pseudo randomized order. Each list contained 64 items that consisted of 16 scenes with agents to the left and 16 mirror images of the same scenes with agents to the right of patients as well as 32 filler items. All participants were tested individually. Each experimental session lasted approximately 30 min.

### Results

In 98.02% of all utterances participants produced agent-first subject-verb-object sentences whereas passive sentences amounted to only 1.98% of the produced utterances. The distribution of the number of responses corresponding to these two utterance types was significantly different from chance, *χ*^*2*^(1) = 885.50, *p *< 0.001. Wilcoxon signed-ranks test showed that the number of produced agent-first sentences did not differ depending on whether the described event scene had an agent positioned on the right (*M* = 15.57, *SD* = 0.82) or on the left (*M *= 15.80, *SD *= 0.92), *z* = − 1.61, *p* = .180, *r *= − 0.21. This result indicates a strong preference for agent-first subject-verb-object sentences in language production. The 1.98% of patient-first passive sentences were produced to describe events with an agent on the left (*M* = 0.20, *SD* = 0.92) and on the right (*M *= 0.43, *SD* = 0.82). Due to the low number of observations, a statistical comparison of produced passive utterances in agent-left versus agent-right pictures was not performed.

**Fig. 3:**
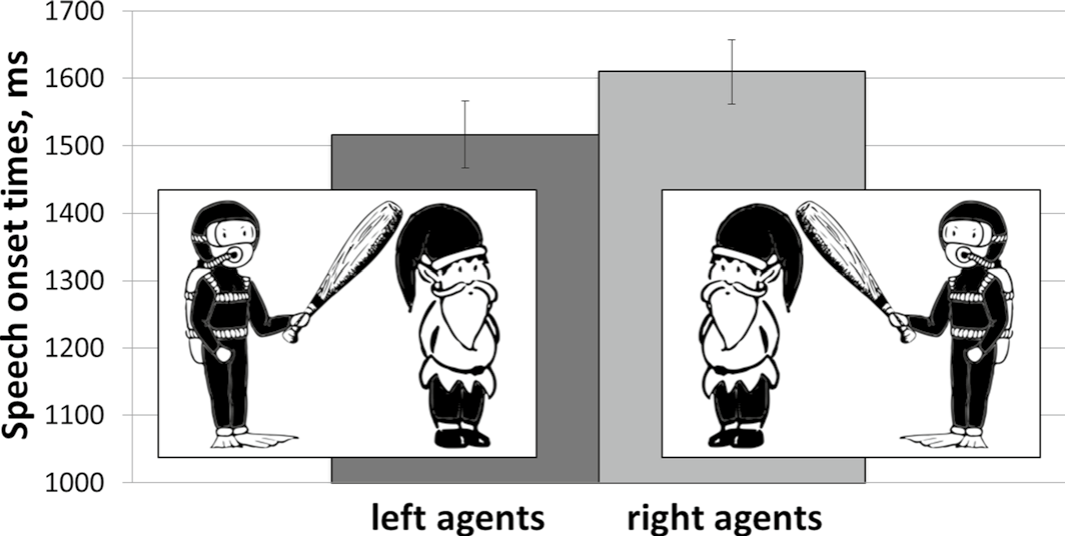
Mean speech onset times of utterances produced for scenes with agents on the left and on the right. The error bars represent standard error of the mean

Paired-samples *t*-tests were used to compare the mean differences between speech onset times when participants described scenes with agents positioned to the left and to the right of patients. The data comprised both active and passive utterance types, no data were excluded for the analyses.^2^ The *t* test on data averaged across participants showed that speech onset times were longer when participants had to describe scenes with agents positioned to the right (*M *= 1609.91, *SD* = 260.60) rather than to the left (*M *= 1516.74, *SD* = 273.97) of patients, *t*(29) = 4.05, *p *< .001, *r* = 0.60 (Fig. 3). The comparison of mean speech onset times averaged across items also showed a significant difference confirming the reliability of the effect, *t*(15) = 3.57, *p* = .003, *r* = 0.68.

### Discussion

The great majority of utterances produced in Experiment 1 were active sentences with a subject-verb-object structure where agents were placed sentence-initially. This observation is in line with both typological and psycholinguistic literature reporting an agent-first placement as a dominant and preferred structure (e.g., Bornkessel-Schlesewsky and Schlesewsky 2009; Kemmerer 2012). The analysis of speech onset times revealed additional costs associated with the production of descriptions for scenes where agents were placed to the right of patients. Such utterances took on average 93 ms longer to be initiated compared to descriptions for scenes where agents were placed to the left of patients. These significant delays in speech production may indicate a mismatch in the alignment of the visually perceived structure of an event and the structure of the planned utterance. Under this assumption, additional costs in sentence production are likely to occur when the spatial ordering of actants in an event scene cannot be directly aligned to the preferred agent-first subject-verb-object structure of the sentence. The shorter speech onset times related to scenes with left-positioned agents indicate a smoother alignment between a visually left-positioned agent and an agent-first sentence structure in language production.

## Experiment 2

Experiment 2 examined whether the preference for left positioned agents manifested in speech onset times in Experiment 1 can be generalized as a phenomenon that affects non-linguistic preferences in non-verbal tasks as well.

### Method

#### Participants

Forty-four students from the University of Cologne took part in the experiment and were offered a candy bar for their participation. The data from eight participants were excluded, as they reported to be non-native speakers of German. Neither of the remaining participants reported to be a native speaker of another language in addition to German. The data from 36 native speakers of German (33 female, 3 male, mean age 24.2 years, *SD* = 1.8) were used for the analyses reported below.

#### Materials and Design

A questionnaire was designed to assess participants’ visual preferences for depicted events. Similar to Experiment 1, participants were presented with black-and-white scenes that depicted two male characters, one of whom performed an action on another one. To present the experimental items on a single sheet of paper, the questionnaire contained nine items, each item included two scenes that were mirror images of the same event placed next to each other together with three response options. Two response options reflected preferences for scenes with either left or right positioned agents and the third indicated no preference. The no-preference option was included to prevent participants from making a forced choice in favor of scenes with agents on the left or on the right as the only alternatives. Items appeared in the questionnaire in a pseudorandomized order. Two questionnaire versions alternated the order of images with left- and right-positioned agents within items to avoid possible influences of viewing order on picture preferences.

#### Procedure

A paper-and-pencil version of the questionnaire was used for testing. Participants were instructed to indicate with a cross one of the two pictures that looked more conventional, natural or in any way better in their opinion. The third provided option was *I have no preference*. No time restriction was applied for completing the questionnaire.

### Results

Friedman’s ANOVA was conducted to compare the effect of visual preferences on the number of responses corresponding to scenes with agents positioned to the left of patients, agents positioned to the right of patients and a *no preference* option. The results showed a non-random distribution of responses indicating preferred scenes, *χ*^*2*^(2) = 21.32, *p *< .001. Wilcoxon signed-rank test was performed to make post hoc comparisons between left-agent and right-agent preference conditions. Participants selected more scenes where agents were depicted to the left (*M *= 5.06, *SD *= 2.48) rather than to the right of patients (*M *= 2.52, *SD* = 1.92) as a preferred response option, *z *= − 3.27, *p *< .001, *r *= − 0.34.

### Discussion

The preference for left-positioned agents observed in sentence production in Experiment 1 was also confirmed by a non-linguistic aesthetic judgment task in Experiment 2. While the task of Experiment 2 did not require an alignment of the visually-depicted actants of the event with syntactic agent-first structures, event scenes depicting agents to the left rather than to the right of patients were more likely to be selected as preferred. This finding suggests that preferences for a particular linear organization of visually represented transitive events may not be dependent on sentence planning, processing or production but also occur in aesthetic judgment tasks not requiring any verbal responses. This supports our interpretation of the data obtained in Experiment 1 that a misalignment between the visual (left-agent) preference in the organization of transitive events and the preference in their linguistic (agent-first) expression may lead to additional costs and delays in language production.

## Experiment 3

Experiment 3 investigated whether the left-agent preference expressed in both linguistic (Experiment 1) and non-linguistic (Experiment 2) tasks in German may be specific for languages with left-to-right writing systems. This experiment aimed to reveal whether preferences for the visuo-spatial position of referents may be shaped by cultural habits related to the reading and writing direction employed in a particular language (as previously suggested by Dobel et al. 2014, Maass and Russo 2003). Therefore, we tested individuals literate in Modern Standard Arabic, which employs a right-to-left direction in its script.

### Method

#### Participants

Forty speakers of Arabic participated in Experiment 3. The data from 3 participants were excluded, as they reported to be non-native speakers of Arabic. Participants had come to Germany as refugees from Arabic-speaking countries, mostly Syria. They were tested in two central accommodation facilities for refugees in the Cologne and volunteered to participate in the experiment. All participants were literate in Modern Standard Arabic and were tested by a native speaker of Arabic who provided all instructions and clarifications in Modern Standard Arabic. The data from 37 native speakers of Arabic (17 female, 20 male, mean age = 34.1 years, *SD* = 9.0) were used for the analyses reported below. Additional reading/writing abilities of these participants in a language with a left-to-right writing system were assessed and accounted for in the analyses.

#### Materials

The questionnaire used in Experiment 2 was translated into Modern Standard Arabic to adapt it for the Arabic-speaking population. The content of the questionnaire remained otherwise unchanged.

#### Procedure

The procedure was the same as described in Experiment 2.

### Results

As in Experiment 2, the computation of Friedman’s ANOVA showed a non-random distribution of responses indicating that participants had visual preferences, *χ*^*2*^(2) = 14.81, *p *< .001. Post hoc pairwise comparison based on Wilcoxon signed-ranks test revealed that the number of selected scenes with agents to the left (*M *= 1.81, *SD* = 1.35) was significantly lower than that of scenes with agents to the right of patients (*M *= 2.49, *SD* = 1.94), *z* = − 1.98, *p* = .049, *r* = − 0.23.

#### Comparisons Between Arabic and German Speakers

**Fig. 4:**
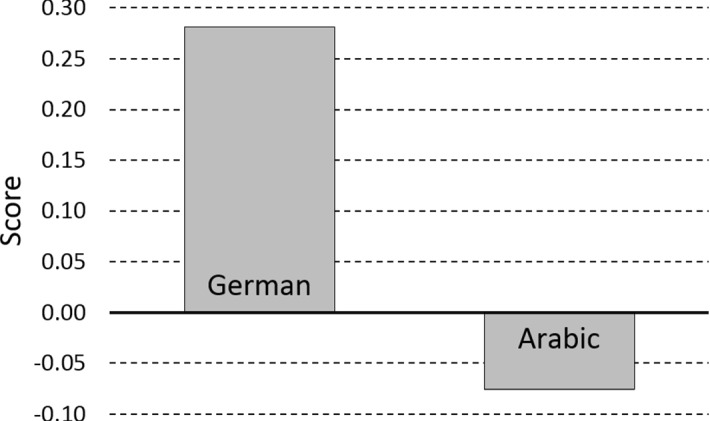
Visual preferences of German and Arabic speakers. Positive and negative scores reflect left- and right-agent preferences respectively

To assess the differences between native Arabic and German participants in their visual preferences, we compared their performance using preference scores (cf. Chokron and de Agostini 2000). These scores represent a difference between left- and right-agent responses (left-agent responses minus right-agent responses divided by the total number of responses), so that positive scores reflect a left-agent preference and negative scores reflect a right-agent preference (Fig. 4).^3^ Welch’s *t*-test was carried out on preference scores averaged across participants (*t*_*1*_) and across items (*t*_*2*_) to compare samples of different sizes. Preference scores of German and Arabic speakers differed significantly, with German speakers manifesting a left-agent (*M* = 0.28) and Arabic speakers a right-agent preference (*M* = − 0.08), *t*_1_(49.36) = 4.37, *p *< .001, *r* = 0.53; *t*_*2*_(14.29) = 3.86, *p* < .001, *r *= 0.71.

To assess the influence of Arabic speakers’ exposure to left-to-right writing systems, we divided them into two groups: a group with (N = 16) and a group without additional reading/writing abilities in a language with a left-to-right writing system (N = 21). We then compared German speakers’ scores with the scores obtained in each of these Arabic speaker groups. Like before, German speakers differed from those of Arabic speakers with reading/writing abilities in a language with a left-to-right writing system (*M* = − 0.05), *t*_1_(49.85) = 4.01, *p *< .001, *r* = 0.49; *t*_*2*_(13.43) = 3.75, *p* = .002, *r *= 0.71, as well as from those without such abilities (*M* = -0.10), *t*_1_(47.13) = 3.99, *p *< .001, *r* = 0.50; *t*_*2*_(16) = 3.60, *p* = .002, *r *= 0.67. At the same time, preference scores of Arabic speakers with and without additional reading/writing abilities in a language with a left-to-right writing system did not differ, *t*_1_(25.67) = − 0.70, *p *= .488; *t*_*2*_(13.40) = − 0.57, *p* < .576.

### Discussion

In contrast to German speakers, when given the same non-verbal task Arabic participants showed a preference for event scenes where agents were depicted to the right of patients. Moreover, the design of our study allowed a direct comparison of visual preferences between German and Arabic participants, showing that the number of left-agent scenes selected as preferred by Arabic participants was significantly lower than that of German speakers. This is consistent with previous findings on the way events containing agents and patients are represented in literate Arabic individuals, suggesting an influence of the reading/writing direction in Modern Standard Arabic (e.g., Maass and Russo 2003). It is worth noting that this visual preference manifested despite the fact that some participants were familiar to an extent with left-to-right writing systems due to their school education of English as a foreign language and their exposure to German script in Germany. The performance of Arabic speakers with and without additional writing/reading abilities in a left-to-right written language did not differ in terms of their visual preferences. Therefore, the experience Arabic participants had with a left-to-right writing system was not sufficient to change their right-to-left preferences. While exposure to a different writing system is likely to affect directional biases once participants become more acquainted with the particular system, it remains an open question how much exposure is necessary in order to elicit a change in participants’ spatial biases. Future studies are needed to address this question. For now, our results suggest that while the directionality bias may manifest in contexts that do not require the involvement of language (i.e., non-verbal tasks), it nevertheless appears to be shaped by the characteristics of the writing system of a language.

## General Discussion and Conclusion

Our study targeted the relationship between the visual organization of transitive events involving an agent and a patient and their representation in language. Unlike previous studies that exclusively focused on links between spatial representations and language comprehension, here we provide first evidence that visual preferences may also affect language production. Experiment 1 indicated that speech onset times of utterances in German speakers were affected by the linear organization of actants in visually represented event scenes, showing a left-to-right directionality bias. While it manifested in the time required for initiating an utterance, this bias did not affect speakers’ syntactic choices. Experiment 2 further investigated the contribution of non-linguistic factors to this bias, suggesting that it may be relatively independent of language planning or processing. Experiment 3 tapped into the nature of this bias showing that its direction in individuals literate in Modern Standard Arabic is reversed compared to those literate in German, indicating that the characteristic differences in the writing systems of these two languages are likely to explain the bias. Based on these findings, we suggest that in describing depicted event scenes participants align a linguistically preferred structure (i.e., agent-first) with a non-linguistic visuo-spatial preference for event depiction (i.e., agent-left in the case of German-speaking participants). When the two directionalities are in opposition, the mismatch is mirrored by delays in language production. Moreover, our data provide evidence that non-linguistic preferences as identified in Experiment 2 may themselves be affected by cultural habits such as the direction of reading and writing. This is suggested by the difference observed between Arabic and German participants who showed opposite preferences for visually depicted events (i.e., agent right of patient in the case of the Arabic participants). While we show that German speakers display longer speech onset times for misaligned events, future studies should examine whether a similar effect can be observed for literate speakers of Arabic. Since participants literate in Modern Standard Arabic show a reversed preference for visual event sequences compared to German-speaking participants (see Experiment 2 and 3), this should also lead to a corresponding difference in sentence production (i.e., faster speech onset times for agent-first sentences when agents are depicted on the *right* as opposed to the left of patients).

Despite differences in script directionality, Arabic and German make use of a similar word order in which the subject is commonly mentioned before the object. In an effort to disentangle effects of word order preferences and writing direction on participants’ spatial biases, Maass et al. (2014) tested speakers of Italian, Arabic, and Malagasy in a drawing experiment and a picture-matching task. While Malagasy makes use of a left-to-right writing system, the predominant word order is verb-object-subject. Interestingly, Malagasy speakers preferred a left-to-right arrangement of characters in a free drawing task, i.e. they preferably placed the agent to the left of patients in line with their writing direction. However, when they had to find a picture that best matched a written sentence in a picture-matching task, they showed the reversed preference (i.e. a right-to-left bias, comparable to speakers of Arabic). While the study of Maass et al. (2014) suggests that writing direction and word order can both affect spatial biases, leading to malleable preferences when the two are in conflict, our study provides some first evidence that, inversely, spatial biases can also affect language production. More research is needed to disentangle the independent contributions and interactions of spatial biases, reading/writing habits and word order in event descriptions.

### The Effects of Agent-First Preferences on Language Production

The predictions made in Experiment 1 considered two factors that are commonly inspected in psycholinguistic research on language production: the type of produced utterance and speech onset latencies. As to the utterance type, we expected German speakers to produce a higher number of agent first subject-verb-object sentences describing scenes where agents were positioned to the left of patients, whereas more patient-first sentences were expected for scenes with agents to the right of patients. However, our results show that participants mainly produced active subject-verb-object sentences, while patient-first sentences were extremely rare. Although the observed reluctance to refrain from active subject-verb-object clauses might be considered unexpected given the relatively free word order in German, this observation is consistent with studies on the influence of perceptual prominence of visual elements on sentence structures. These studies conclude that language users tend to keep their preferred word order instead of changing it to fit a perceptually salient element into subject position (Bock et al. 2004). In contrast to German speakers, previous research has shown that English speakers are more likely to produce a passive sentence when visual attention is drawn to the patient of a depicted action (Gleitman et al. 2007; Myachykov et al. 2018). This suggests that the propensity to produce a passive in scene-description tasks displays cross-linguistic variation. German speakers’ structural choices seem to be far less influenced by visual cues, although visual cueing is as effective in drawing initial looks to a depicted patient as in English (Esaulova et al., in press) and passive utterances appear with a frequency comparable to English in language production corpora (5–6% passives in English (Roland et al. 2007), 7–9% in German (Bader and Häussler 2010)).

At the same time, speech onset latencies confirmed the expected differences between scenes depicting agents to the right as opposed to those depicting agents to the left of patients. Specifically, speech onset times indicate that speakers were quicker in initiating an utterance to describe scenes with agents depicted to the left than to the right of patients. This finding suggests that for German speakers scenes are easier to process when agents are placed to the left of patients (also see Dobel et al. 2007a for converging evidence). As a consequence, a left-to-right arrangement of event characters also leads to a speeded linguistic encoding of the event. Following Chatterjee et al. (1999) who observed similar preferences in tasks involving language comprehension, depictions of event scenes may thus be conceived of as representations of argument structures where depicted characters represent thematic roles of agents and patients (also see Dobel et al. 2007a for a related argument).

Moreover, our finding that the visual organization of event scenes can impact on sentence production has methodological implications for other psycholinguistic studies that use picture description tasks. As our experiments demonstrate, the spatial orientation of actants not only reveals visual preferences but also influences sentence production. Therefore, we emphasize the importance of controlling it as a factor rather than counterbalancing left- and right-occurring actants in experimental stimuli.

### Biases in Visual Event Representations

Whether the observed bias for left-to-right directionality necessarily relies on language use was tested in Experiment 2. In a non-verbal picture-preference task, German speakers preferred scenes with agents depicted to the left of patients to pictures showing agents on the right of patients, thus revealing the same bias that surfaced in speech onset times in Experiment 1. Unlike previous studies, we made use of a simple aesthetic judgment task not involving any potentially biasing motor activity (such as drawing). Furthermore, while previous tasks explicitly asked participants to match pictures to sentences (e.g. Maass et al. 2014), the preference task employed in the current study was not designed to link visual scenes to language. Still, the preference for event scenes depicting agents to the left of patients was observed both when participants were asked to describe the scenes (Experiment 1) and when they were instructed to simply select a “better” picture (Experiment 2). This may be the result of an analogy between the spatial linearity of visual representations and the structural organization of speech. The location of agents relative to patients and the visually represented left-to-right movement of an action appear to be isomorphic to the linear placement of agents in sentence-initial positions. In this way, preferences expressed in both verbal and non-verbal tasks manifest an overlap between visual and linguistic representations of transitive events in terms of spatial and temporal sequences. Our results support the idea that even non-verbal events tend to be sequenced in an order where agents precede patients (at least in languages that favor subject-object word order, see arguments presented above). Here we could show that the visual preference for events to be structured from left to right starting with an agent coincided with participants’ verbal preference to place agents first in an utterance.

### Modulation of Preferences Via Reading/Writing Habits

Previous findings have indicated that visual preferences may be due to the writing system of a particular language and the visual habits associated with it (e.g., Maass and Russo 2003; Dobel et al. 2007b, Dobel et al. 2014; Butler et al. 2014). In accordance with these findings, we could show that a visual preference for left-positioned agents evident in German participants who employ a left-to-right script was reversed in Arabic participants using the right-to-left script of Modern Standard Arabic, thus contributing to the existing evidence on the link between reading/writing direction in a language and visual preferences. Visual preferences observed in German participants in our study were stronger than those of Arabic speakers. This finding, however, is in line with Maass and Russo (2003) who asked Italian and Arabic participants to draw scenes representing sentences like “The girl pushes the boy”. The authors report an asymmetry similar to that found in our study, with a left-to-right visual bias demonstrated by Italians being stronger than its reversal in Arabs. Following Chatterjee et al. (1999), the authors provide some evidence that hemispheric specialization might explain the different strength of the bias in the two groups. They suggest that directing attention from left to right—a process unrelated to language and driven by the left hemisphere—may either match or mismatch the direction of speakers’ visual biases and thus modify their magnitude. Nevertheless, Maass and Russo (2003) indicate that the weight of such influences on visual preferences is by far less significant than that of scanning habits shaped by reading/writing experiences (for additional evidence see also Dobel et al. 2007b). Reading from left to right versus right to left may shape people’s visual routines in opposite directions, thereby affecting their respective preferences for depictions of event scenes. Importantly, our results show that cross-cultural differences in spatial biases are effective even when simple non-linguistic tasks are used.

Reading habits can also influence the way participants represent temporal events more generally. For instance, when asked to arrange temporal sequences (e.g., images of a person depicted at varying ages from young to old), English and Hebrew speakers who are exposed to different writing directions in their culture also ordered the sequences in correspondingly different ways (Fuhrman and Boroditsky 2010). While English-speakers started with the ‘earliest’ stage of a temporal sequence at the leftmost position followed by later stages, the reverse was true for Hebrew speakers, demonstrating that the way a temporal sequence is transferred to the spatial domain is dependent on reading and writing habits. While participants in our Experiments 2 and 3 only had to indicate preferences for a particular picture, they still had to match the temporal and spatial unfolding of the visually represented event in order to properly interpret the presented images. Like in previous studies (e.g., Maass and Russo 2003; Dobel et al. 2007b, Dobel et al. 2014; Butler et al. 2014), this matching appeared to be sensitive to the reading and writing directionality, providing further evidence that reading habits can affect the way people encode events.

### The Alignment of Verbal and Non-verbal Preferences

While in our study we only manipulated the visuo-spatial position of referents in scenes, we observed clear preferences for agents to appear either on the right or on the left depending on the language participants were literate in: those literate in German preferred left-positioned agents, while participants literate in Modern Standard Arabic rather preferred right-positioned agents. Despite the apparent reversal of preferences in these two groups of language users, the mechanism underlying these preferences may be the same if scenes are thought of as linear representations of syntactic structures in sentences. In fact, both preferences may reflect an alignment between the visual linearization and the syntactic structure where an agent/subject precedes a patient/object—a predominant pattern in both German and Arabic languages. While future studies should investigate the possible impact of differences in word order more closely (i.e. subject-object vs. object-subject), our results suggest the following: the predominant subject-object pattern in both German and Arabic seems to be reflected by left-positioned agents in a language system with a left-to-right script, such as German, and by right-positioned agents in a language with a right-to-left script, such as Modern Standard Arabic. Interestingly, the mapping between the depicted referents and their syntactic functions becomes apparent in both, linguistic tasks that require the activation of the corresponding syntactic structures (Experiment 1) and non-linguistic visual-preference tasks (Experiments 2 and 3). These findings indicate that the visual and verbal preferences, although independent from each other as belonging to different modalities, may nevertheless closely interact, mapping onto one another.

## Conclusion

Overall, we have shown that German-speaking participants prefer visual scenes in which agents are positioned to the left of patients, even when a non-linguistic preference task is used. We have also demonstrated that this non-linguistic visuo-spatial preference seems to have an impact on sentence production. Unlike previous studies that exclusively focused on links between spatial representations and language comprehension, here we provide the first evidence that visual preferences may also affect language production. In particular, German-speaking participants were slower in producing agent-first utterances when their left-to-right preference for actants in a visual scene was violated. This finding suggests that participants may align a linguistically preferred structure (i.e., agent-first) with a non-linguistic visuo-spatial preference involving the depiction of events (i.e., agent-left in the case of German-speaking participants). However, when the two directionalities are in opposition, this mismatch is mirrored by delays in language production. While our results point to links between non-linguistic preferences of event scenes and ease of production, they likewise indicate that non-linguistic preferences themselves may be affected by cultural habits such as directions of reading and writing. Thus, when tested in the exact same preference task, Arabic participants differed from German participants by showing the opposite preference for visually depicted events.

Our results reveal that non-linguistic factors such as the visual organization of events can impinge on language production. At the same time, the visual preferences for the serialization of events may in turn be affected by cultural habits such as reading and writing direction. Taken together, our findings suggest that the relationship between visual and linguistic structure might be more complex than previously attested. Broadening the scope to cross-cultural variation allows for capturing some novel aspects of this relationship, thereby providing insights into the complex interactions between language and the visual world.

## Footnotes

1 Lemma frequencies were collected using CELEX2 corpus (Baayen et al. 1995).

2 Analyses on data with passive utterances excluded were carried out but are not provided here, as their results did not differ in any important way from the ones reported below.

3 This approach allows to account for the fact that Arabic participants produced more no-preference responses than German participants, *U* = 247, *p* < .001. This, however, does not seem to be due to the familiarity of participants with only one directionality of a writing system (German speakers) rather than both right-to-left and left-to-right directionalities (most Arabic speakers), as Arabic participants with and without additional writing/reading skills in a left-to-right written language did not differ with regard to their no-preference responses, *U* = 156.5, *p* = .734.
